# Refractive Index Sensors Based on Long-Period Grating in a Negative Curvature Hollow-Core Fiber

**DOI:** 10.3390/s21051803

**Published:** 2021-03-05

**Authors:** Hanna Izabela Stawska, Maciej Andrzej Popenda

**Affiliations:** Department of Telecommunications and Teleinformatics, Wroclaw University of Science and Technology, 50-370 Wroclaw, Poland; maciej.popenda@pwr.edu.pl

**Keywords:** microstructured fibers, hollow-core fibers, antiresonant fibers, optical fiber design, optical fiber sensors, long-period fiber gratings, microstructured optical fiber long-period gratings

## Abstract

Long-period optical fiber gratings (LPGs) are one of the widely used concepts for the sensing of refractive index (RI) changes. Negative curvature hollow-core fibers (NCHCFs), with their relatively large internal diameters that are easy to fill with liquids, appear as a very interesting medium to combine with the idea of LPGs and use for RI sensing. However, to date, there has been no investigation of the RI sensing capabilities of the NCHCF-based LPGs. The results presented in the paper do not only address this matter, but also compare the RI sensitivities of the NCHCFs alone and the gratings. By modeling two revolver-type fibers, with their internal diameters reflecting the results of the possible LPG-inscription process, the authors show that the fibers’ transmission windows shift in response to the RI change, resulting in changes in RI sensitivities as high as −4411 nm/RIU. On the contrary, the shift in the transmission dip of the NCHCF-based LPGs corresponds to a sensitivity of −658 nm/RIU. A general confirmation of these results was ensured by comparing the analytical formulas describing the sensitivities of the NCHCFs and the NCHCF-based LPGs.

## 1. Introduction

Optical fibers are considered an interesting solution for the design of refractive index sensors (RISs) due to their compact size, immunity to electromagnetic interference, rapid response and high sensitivity. Fiber gratings are a particularly common device for the purpose of sensing, with their variation known as long-period gratings (LPGs), fabricated by introducing a longitudinal periodic refractive index and/or structural modulation along an optical fiber [1]. The modulation, typically with a period in the range of hundreds of micrometers, induces resonant coupling between the fundamental and higher order modes—a mechanism well described by the phase-matching condition [2,3]:

(1) $$N\lambda_{N}^{\left(\mathrm{LPG}\right)}=\left(n_{eff}^{f}-n_{eff}^{ho}\right)\Lambda$$

where $n_{eff}^{f}$ and $n_{eff}^{ho}$ are the effective refractive indices of the fundamental and high-order modes, respectively, *N* is the order of the resonance, $\lambda_{N}^{\left(\mathrm{LPG}\right)}$ is the *N*-th resonant wavelength and *Λ* is the period of the grating. However, a more rigorous approach additionally considers self-coupling of the same order modes between the consecutive LPG grating regions, resulting in a modified phase-matching condition [4,5]:

(2) $$\left(\beta_{01}\left(\lambda \right)+s_{0}\zeta_{01,01}\left(\lambda \right)\right)-\left(\beta_{\nu j}\left(\lambda \right)+s_{0}\zeta_{\nu j,\nu j}\left(\lambda \right)\right)=\frac{2\pi N}{\Lambda }$$

where $\zeta_{01,01}\left(\lambda \right)$ and $\zeta_{\nu j,\nu j}\left(\lambda \right)$ are the LP01 and LPνj mode self-coupling coefficients, respectively, $\beta_{01}\left(\lambda \right)$ and $\beta_{\nu j}\left(\lambda \right)$ are the LP01 and LPνj mode propagation constants $\left(\beta \left(\lambda \right)=n_{eff}\frac{2\pi }{\lambda }\right)$, while *s*_0_ coefficient is the zero-frequency Fourier component of the LPG. The main problem associated with the optical fiber refractometers is that they achieve their highest sensitivity for *n* values of 1.4 and higher, as mentioned by Guo et al. [6] and recently confirmed in the review work on the topic of LPG and Mach–Zehnder interferometers by Eftimov et al. [7]. Indeed, one can find a relatively recent report on a conventional, SMF-based (single-mode fiber) LPG with sensitivities of approximately 800 nm/RIU in the RI range of 1.33–1.4, while the same LPG for the RI above 1.4 achieves sensitivities in the order of thousands [8]. Higher sensitivities, ranging from 2000 nm/RIU [9] up to even 20,000 nm/RIU [10], can be obtained. However, they additionally require the use of technologies such as thin-film coating, precise etching of the fibers, working in the dispersion turning point of the LPG or others, described in more detail in the review works [7,11]. An alternative way to increase the sensitivity of LPG RI sensors is to use microstructured optical fibers (MOFs), which contain a unique type of cladding consisting of an array of air holes. Currently, we can roughly distinguish between two main types of MOFs: the photonic-crystal (PCF) and the antiresonant (ARF) fibers [12,13]. In the case of PCF-based LPGs, there have been a number of reports describing both their fabrication and optical characteristics [14,15,16], as well as their environmental sensing features [17]. The matter of using PCF-based LPGs for RI sensing has also been investigated, with the reported sensitivities ranging from 1500 [18] to 2400 nm/RIU [19] at RI below 1.4. Another important type of PCF—the hollow-core photonic bandgap fibers (HCPBFs)—has also been used as an RI sensor, exhibiting a blue wavelength shift (towards shorter wavelengths) of 280 nm when the refractive indices changed from 1.33 to 1.39, resulting in an approximate sensitivity (absolute value) of 4667 nm/RIU [20]. HCPBF-based LPGs have also been filled with liquids, but due to the liquids’ RI values being very close to or higher than the RI of the fiber, no shift in the LPG resonant peak was observed [14]. Unfortunately, PCF sensors (both solid-core and hollow-core) exhibit a major drawback associated with the rather cumbersome filling/infiltration of the small (<1 µm) holes [21,22]. ARFs, on the contrary, with their most common variation known as NCHCFs (negative curvature hollow-core fibers), are easier to fill due to the fact that their core and capillary diameters are larger [23], and they have already been used extensively in different experiments [24,25,26]. The wave-guiding mechanism of the NCHCFs is described by the inhibited coupling (IC) model [13]. However, their transmission bands can be determined using the antiresonant-reflective optical waveguide (ARROW) model [12], which allows to calculate the antiresonant wavelength (i.e. the central wavelength of the fiber’s low-loss region) from the following equation [27]:

(3) $$\lambda_{m}^{\left(\mathrm{AR}\right)}=\frac{4t}{2m+1}\sqrt{n_{g}^{2}-n_{l}^{2}}$$

where *t* is the capillary wall thickness, *m* is the order of the antiresonant wavelength $\lambda_{m}^{\left(\mathrm{AR}\right)}$, *n_g_* is the refractive index of the glass (or, more generally speaking, the material of the fiber’s microstructure) and *n_l_* is the refractive index of the medium filling the microstructure of the fiber. The research which is possibly the closest to that presented in this paper was conducted by Wei et al., who numerically investigated the possibility of using a liquid-filled NCHCF as a temperature sensor, showing that such fibers can be used to design sensors with RI sensitivities as high as −3230 nm/RIU [26]. The authors of the presented paper conducted an experiment with the Kagomé-style NCHCFs, showing that the transmission windows shift in response to the different refractive index of the liquid filling the fibers, with the measured sensitivities (absolute values) being approximately 2870 nm/RIU [28]. Nevertheless, to date, the matter of using NCHCF-based LPGs has not been examined thoroughly. Huang et al. managed to convert the NCHCF into an in-line fiber filter [29] by locally heating an NCHCF with a CO_2_ laser and creating a deformation in the NCHCF’s microstructure. In our opinion, a very similar method can be used to write an LPG into an NCHCF.

In this work, a sensor for the detection of the refractive index of aqueous samples, based on an NCHCF with an inscribed LPG and without it, is numerically and analytically studied. The comparison of both sensors’ performance leads to an unexpected and, to the authors’ best knowledge, unpublished result of the NCHCF-based LPG sensor’s RI sensitivity being significantly lower than the sensitivity of the unmodified NCHCF. By analyzing the formulas describing the fiber’s and grating’s sensitivities, the authors draw the conclusion that obtaining an LPG based on an NCHCF with its intended RI sensitivity higher than the sensitivity of the fiber alone is a very difficult task.

## 2. Results and Discussion

### 2.1. NCHCF and NCHCF-Based LPG—Models and Diameters

**Figure 1:**
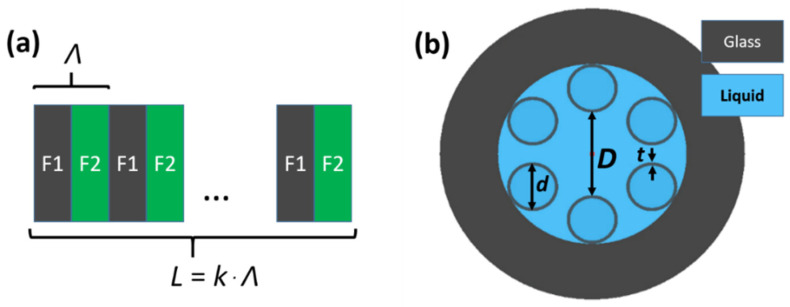
(**a**) Model of the investigated long-period fiber grating (LPG) formed as a composition of the alternating segments of fibers F1 and F2. The total length *L* of the LPG is equal to the grating period *Λ* times *k*, where *k* = 60 is the number of repetitions of a single period. (**b**) Cross-section of the negative-curvature hollow core fiber (NCHCF). The marked dimensions *D*, *d* and *t* were used to determine the optical parameters of the fiber and LPG; their values for the F1 and F2 fibers can be found in the main text below.

The LPGs were modeled as a composition of two segments of different NCHCFs, named F1 and F2, and repeated *k* times, as depicted in Figure 1a. The total length of the LPG was *L* = *kΛ*, where *k* = 60 is the number of repetitions of the periods and *Λ =* 340 µm is the period of the LPG. This resulted in the total length of the LPG being *L* = 2.04 cm, allowing us to obtain a strong transmission dip at *λ*_LPG_ = 700.5 nm and refractive index of the liquid *n*_l_ = 1.33; the choice of *Λ* and *λ*_LPG_ is described in more detail in Section 2.3. In Figure 1b, a cross-section of the NCHCF used in this study is presented. The structure is defined by the following parameters: core diameter *D*, capillary wall thickness *t* and outer capillary diameter *d*. The dark gray color depicts glass regions and the blue color depicts liquid regions.

A possible application that would draw particular attention towards the designed sensor would be the detection of RI changes in biological samples. Optical methods in biochemical and medical sciences, in general, use wavelengths from the VIS-NIR range [30,31,32,33]. Although the use of longer wavelengths (above 1000 nm), especially for the purpose of imaging, is currently on the rise [31,34,35], the problem of water absorption limits the sample thickness to a maximum of a few millimeters. Since the proposed sensor’s working principle requires the filling of the fiber with a liquid sample, the predicted optical lengths will be around ~5 cm, making the water-induced attenuation of the signal too high for the sensor to be reliable for use with *λ* > 1000 nm. On the other hand, shorter wavelengths are much more transparent for water-based samples, and the authors decided to design both the F1 and F2 fibers’ 1st antiresonant windows (i.e., the window of the fiber’s lowest attenuation) in the 600–1000 nm range, assuming the refractive index of the initial sample (pure water) to be 1.33. Designing the fibers in such a manner ensures the eventual sensor’s RI sensing capability to cover the RI range of 1.33 up to even 1.42, since one will observe a blue shift in the antiresonant wavelengths, according to Equation (3). Finally, the following geometrical parameters were chosen for the two fibers: *D* = *D_F1_* = 20 µm, *d* = *d_F_*_1_ = 7 µm and *t* = *t_F_*_1_ = 1 µm for fiber F1 and *D* = *D_F_*_2_ = 20.8 µm, *d* = *d_F_*_2_ = 6.8 µm and *t* = *t_F_*_2_ = 1.1 µm for the fiber F2. The diameters of the F2 fiber section are derived from the fact that due to the glass viscosity, under CO_2_ laser radiation (or another type of heat processing of the fiber), the capillaries will shrink, while their wall thickness and core diameter will increase. An important feature of introducing such a perturbation is that it can be expected to be fairly uniform [29].

### 2.2. NCHCF—Optical Parameters and Sensitivity to the Refractive Index Changes

**Figure 2:**
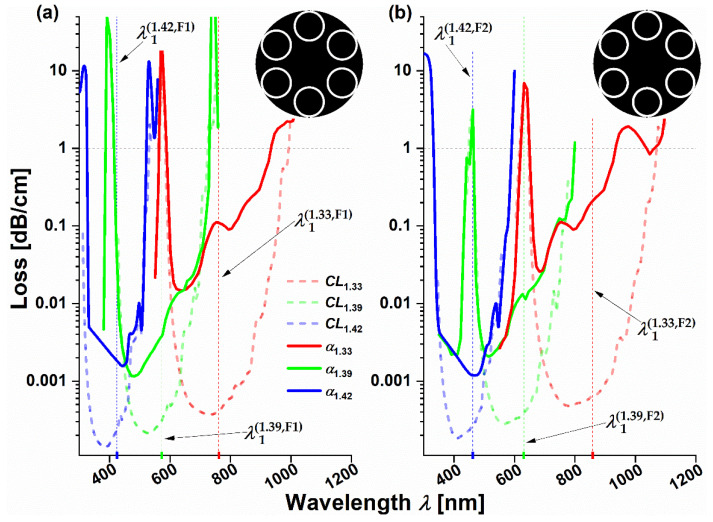
Loss spectra of fibers F1 (**a**) and F2 (**b**) versus the wavelength (*λ*) for 3 different values of the refractive index of aqueous liquids filling their microstructures: 1.33, 1.39 and 1.42. The confinement losses of both fibers (*CL*, dashed lines) are shown in order to better mark the presence of the antiresonant windows. The total losses (*α*, solid lines) of both fibers additionally account for the water absorption. The red, green and blue ticks and dotted lines at the X-axis (additionally indicated by the black arrows) mark the central wavelengths of the first-order (*m* = 1) antiresonant bands of F1 and F2, calculated at *α* = 1 dB/cm for each refractive index (RI). The respective values of $\lambda_{1}^{\left(1.33,F1\right)}$, $\lambda_{1}^{\left(1.39,F1\right)}$, $\lambda_{1}^{\left(1.42,F1\right)}$ are 761, 572 and 424 nm for the fiber F1, while for the fiber F2 $\lambda_{1}^{\left(1.33,F2\right)}$, $\lambda_{1}^{\left(1.39,F2\right)}$ and $\lambda_{1}^{\left(1.42,F2\right)}$ are 859, 631 and 462 nm. The insets in the upper-right corner of each spectrum show the cross-sections of the microstructures of F1 and F2, with F2 having slightly thicker capillary walls and a larger core compared to the fiber F1.

Figure 2a,b shows the calculated loss spectra of the fundamental mode LP01 for 3 different values of the liquid’s refractive index for fibers F1 and F2, respectively. Antiresonant wavelengths, marked on the graph’s X-axes by the red, blue and green ticks and dotted lines of the same colors, were calculated from the numerical data as the average of two wavelengths at a loss of 1 dB/cm for the corresponding RI. During the calculations, the dispersion and absorption of both silica glass *n_g_* and water *n_l_* (SiO_2_ and H_2_O Palik models from Lumerical^®^ materials library) were taken into account. Although the water absorption increases the total loss, especially for the longer wavelengths, the overall shape of the antiresonant windows is maintained for both fibers (for the dispersion curves of liquids with *n* > 1.33 and the loss spectra of pure water, please see Appendix A).

According to Equation (3), $\lambda_{m}^{\left(\mathrm{AR}\right)}$ will shift with the change in the refractive index *n_l_*. Indeed, in case of the F1 fiber for the *m* = 1 resonance, $\lambda_{m}^{\left(\mathrm{AR}\right)}$ shifts from 761 to 572 nm and then to 424 nm for the consecutive refractive index values (*n_l_* = 1.33, 1.39 and 1.42), resulting in a total F1 wavelength shift of Δ*λ_F_*_1_ = −337 nm, while the fiber F2 exhibits an even larger shift, from 859 to 462 nm, which gives Δ*λ_F_*_2_ = −397 nm. The presented results are in good agreement with those obtained from a derivative (after *n_l_*) of Equation (3) which, after substitution of *dλ_m_* and *dn_l_* by Δ*λ_m_* and Δ*n_l_*, takes the following form:

(4) $$\Delta \lambda_{m}^{\left(AR\right)}=\frac{-4tn_{l}}{\left(2m+1\right)\sqrt{n_{g}^{2}-n_{l}^{2}}}\Delta n_{l},$$

Knowing that Δ*n_l_* = 1.42 − 1.33 = 0.09, *n_g_* = 1.455, *m* = 1, *n_l_* = 1.33 and considering the F1 fiber, for which *t* = 1 µm, we obtain Δ*λ_F_*_1_ = −270 nm. Of course, the presented results are only a crude approximation of the differentiation, which explains the ~70-nm difference between the numerical and analytical results. However, Equation (4) allows the description of the refractive index sensitivity of the NCHCFs, *S*_Δ*n*NCHCF_, by a simple formula [26]:

(5) $$S_{\Delta n\mathrm{NCHCF}}=\frac{\Delta \lambda_{m}^{\left(\mathrm{AR}\right)}}{\Delta n_{l}}=\frac{-4tn_{l}}{\left(2m+1\right)\sqrt{n_{g}^{2}-n_{l}^{2}}},$$

Numerically obtained wavelength shifts correspond to RI sensitivities *S*_Δ*n*NCHCF_ of −3744 and −4411 nm/RIU for $\lambda_{1}^{\left(\mathrm{AR}\right)}$ for F1 and F2 fibers in the RI range of 1.33 to 1.42, respectively. Reducing the upper RI limit to 1.39 causes the sensitivities to drop to −3150 (fiber F1) and −3800 nm/RIU (fiber F2), which is still a promising result, suggesting the high potential of the NCHCFs for the RI sensing of water-based samples. An important aspect to note is that biological samples are rich in organic compounds, such as amino acids, proteins and lipids, both absorbing and emitting light in the UV–VIS region, especially in the 300–500 nm range [36]. Since the antiresonant bands of fibers F1 and F2 cover this exact wavelength range for *n* = 1.39 to *n* = 1.42, one should take additional precautions during the measurements for such high refractive index values in order to correct for any possible signal distortions, resulting from both the absorption and fluorescence of the samples.

### 2.3. NCHCF-Based LPGs—Optical Parameters and Sensitivity of the Grating Wavelength Dip to the Refractive Index Changes

**Figure 3:**
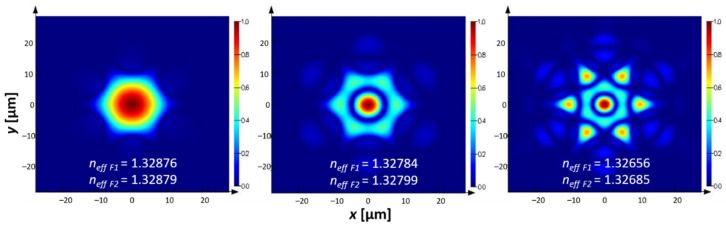
Mode profiles of the first 3 fundamental modes LP01, LP02 for the designed fibers, at the core refractive index *n* = 1.33. The shape of the presented mode profiles is the same for both F1 and F2, and the small difference in the diameters of both fibers does not influence this. The circular symmetry of all modes is clearly visible, satisfying the condition for efficient coupling between them [5]. Their effective refractive indices for the F1 and F2 fibers are also additionally presented.

**Figure 4:**
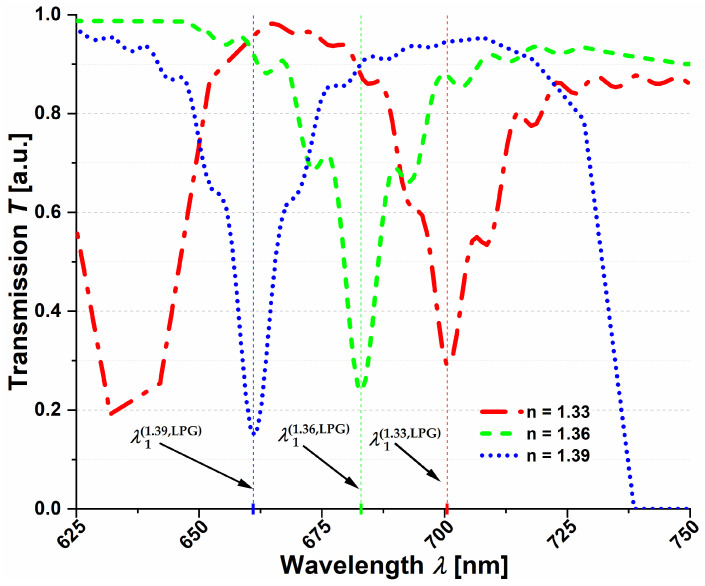
Transmission windows of the NCHCF-based LPG for 3 consecutive values of the liquid’s refractive index *n*. The corresponding first-order (*N* = 1) LPG transmission dips are described as $\lambda_{1}^{\left(1.33,\;\mathrm{LPG}\right)}$, $\lambda_{1}^{\left(1.36,\;\mathrm{LPG}\right)}$ and $\lambda_{1}^{\left(1.39,\;\mathrm{LPG}\right)}$ and their values are 700.5, 683 and 661 nm, respectively. One can also notice the shift in the dips’ positions relative to the fiber’s transmission window, starting closer to the window’s rising edge at *n* = 1.33 and ending near its falling edge at *n* = 1.39. The rise in transmission for wavelengths close to 625 nm and *n* = 1.33 results from the superposition of loss characteristics of fibers F1 and F2 and should not be confused with the appearance of the second-order antiresonant window.

According to the coupled mode theory, uniform refractive index perturbations within the fiber core produce LP01-to-LP0*k* mode interactions [5]. The considered LPG was designed in the vicinity of 700 nm, which is the beginning of the transmission window of the superposition of the antiresonant bands of F1 and F2. Using Lumerical^®^ Mode Solutions software, we calculated the effective refractive indices (*n_eff_*) and mode profiles for the first three LP0*k* modes of the F1 and F2, as presented in Figure 3. For further calculations, we assumed *Λ* = 340 µm, which was expected to allow for an efficient coupling from LP01 to LP03 mode at *λ* = 700.5 nm and *n* = 1.33. There were two main reasons behind the selection of the LP03 mode: firstly, its loss was significant enough (approximately 15.4 dB/cm, compared to 0.85 dB/cm for the LP02 mode) to achieve good grating efficiency at the desired wavelength; the second reason was the small difference in *n_eff_* between the LP01 and LP03 modes, resulting in technically feasible grating periods. Transmission spectra (obtained by means of the Lumerical^®^ Eigenmode Expansion (EME) engine) of the LPG for three different refractive index values are presented in Figure 4. One can clearly observe the appearance of the grating transmission dip, located at $\lambda_{1.33}^{\left(\mathrm{LPG}\right)}$ = 700.5 nm for *n*_1_ = 1.33, and its shifting for the consecutive RI values of 1.36 and 1.39, for which $\lambda_{1.36}^{\left(\mathrm{LPG}\right)}$ = 683 nm and $\lambda_{1.39}^{\left(\mathrm{LPG}\right)}$ = 661 nm. An interesting observation is that the dip itself shifts more slowly than the transmission windows of the NCHCF. When comparing the dip’s position for the three consecutive values of RI, one sees that it appears close to the rising edge, in the middle and finally close to the falling edge of the transmission window. The differences between the dip wavelengths are 17.5 and 22 nm (towards the shorter wavelengths) when changing RI from 1.33 to 1.36 and from 1.36 to 1.39, respectively, resulting in *S*_Δ*n*LPG_ = −658 nm/RIU. Comparing this result with the sensitivities of F1 and F2 in the same RI range (−3150 and −3800 nm/RIU), one sees that the LPG sensitivity is over five-times lower than the sensitivity of the fibers F1 and F2 alone. Additionally, since the transmission windows of both fibers shift faster than the LPG dips, the total refractive index range covered by the LPG is smaller when compared to the bare fibers. The results above are intriguing since the expectations were that the already very promising RI sensing capabilities of the NCHCFs would be further enhanced by inscribing an LPG onto them.

### 2.4. Analytical Comparison of the NCHCF and NCHCF-Based LPG Refractive Index Sensitivities

In order to describe the observed differences in the RI sensitivities of NCHFCs and LPGs in a more general, analytical manner, we decided to compare Equation (4) with a derivative (after *n_l_*) of Equation (1). To do this, we first used the Marcatili–Schmeltzer (MS) tubular waveguide model to substitute the *n_eff_* in Equation (1) [37]:

(6) $$n_{eff_{\nu j}}=\sqrt{n_{l}^{2}-\frac{u_{\nu j}^{2}}{r^{2}k_{0}^{2}}},$$

where *r* is the fiber core radius, *n_l_* is the refractive index of the core (in our case, the RI of the liquid filling the fiber’s core and structure), *k*_0_ = 2π/λ is the wavenumber and *u_νj_* is the *νth*-zero of the *jth*-order Bessel function of the first kind. After substituting (6) to (1) and differentiating after *n_l_*, we obtain:

(7) $$d\lambda_{N}^{\left(\mathrm{LPG}\right)}=\frac{\Lambda }{N}\left(\frac{n_{l}}{\sqrt{n_{l}^{2}-\frac{u_{01}^{2}}{r_{1}{}^{2}k_{0}^{2}}}}-\frac{n_{l}}{\sqrt{n_{l}^{2}-\frac{u_{\nu j}^{2}}{r_{2}{}^{2}k_{0}^{2}}}}\right)dn_{l},$$

Similarly as in (4), we substituted $d\lambda_{N}^{\left(\mathrm{LPG}\right)}$ by $\Delta \lambda_{N}^{\left(\mathrm{LPG}\right)}$ and *dn_l_* by Δ*n_l_* in order to determine the formula describing *S*_Δ*n*LPG_:

(8) $$S_{\Delta n\mathrm{LPG}}=\frac{\Delta \lambda_{N}^{\left(\mathrm{LPG}\right)}}{\Delta n_{l}}=\frac{\Lambda }{N}\left(\frac{n_{l}}{\sqrt{n_{l}^{2}-\frac{u_{01}^{2}}{r_{1}{}^{2}k_{0}^{2}}}}-\frac{n_{l}}{\sqrt{n_{l}^{2}-\frac{u_{\nu j}^{2}}{r_{2}{}^{2}k_{0}^{2}}}}\right),$$

Because Equations (5) and (8) represent the change in the antiresonant/dip wavelength to the change in refractive index of the NCHCF and NCHCF-based LPG, respectively, the final step in our analysis was the investigation of the following function:

(9) $$\begin{array}{cc} F & =S_{\Delta n\mathrm{NCHCF}}-S_{\Delta n\mathrm{LPG}}\\ \; & =\left[\frac{-4tn_{l}}{\left(2m+1\right)\sqrt{n_{g}^{2}-n_{l}^{2}}}-\frac{\Lambda }{N}\left(\frac{n_{l}}{\sqrt{n_{l}^{2}-\frac{u_{01}^{2}}{r_{1}{}^{2}k_{0}^{2}}}}-\frac{n_{l}}{\sqrt{n_{l}^{2}-\frac{u_{\nu j}^{2}}{r_{2}{}^{2}k_{0}^{2}}}}\right)\right], \end{array}$$

Function *F* allows the determination of the difference between the sensitivities *S*_Δ*n*NCHCF_ and *S*_Δ*n*LPG._ In general, *S*_Δ*n*LPG_ can be either positive or negative, meaning that the observed shift in the grating’s transmission dip will occur towards the longer (positive) or shorter (negative) wavelengths. On the other hand, *S*_Δ*n*NCHCF_ is always negative due to the presence of the minus sign (see Equation (5)). In order to determine whether the sensitivity of the LPG is higher than the sensitivity of the NCHCF, two separate cases of Equation (9) (both presented in Table 1) should be analyzed.

#### 2.4.1. Case 1—*S*_Δ*n*NCHCF_ < 0, *S*_Δ*n*LPG_ > 0 and *F* < 2*S*_Δ*n*NCHCF_

When considering case 1, we can limit the analysis of function *F* to the analysis of the *S*_Δ*n*LPG_ > 0 condition itself. Due to the fact that *n_l_*, *Λ* and *N* are all positive values, *S*_Δ*n*LPG_ > 0 can further be simplified to the following inequality:

(10) $$\left(\frac{1}{\sqrt{n_{l}^{2}-\frac{u_{01}^{2}}{r_{1}{}^{2}k_{0}^{2}}}}-\frac{1}{\sqrt{n_{l}^{2}-\frac{u_{\nu j}^{2}}{r_{2}{}^{2}k_{0}^{2}}}}\right)>0$$

In order to solve (10), *r*_2_ was assumed to be the only unknown variable while the rest are parameters. As a result, the following solutions were obtained:

(11) $$n_{l}<-\frac{u_{01}}{k_{0}r_{1}}\wedge \left(r_{2}<-\frac{r_{1}u_{\nu j}}{u_{01}}\vee \;r_{2}>\frac{r_{1}u_{\nu j}}{u_{01}}\right)$$

(12) $$n_{l}>\frac{u_{01}}{k_{0}r_{1}}\wedge \left(r_{2}<-\frac{r_{1}u_{\nu j}}{u_{01}}\vee \;r_{2}>\frac{r_{1}u_{\nu j}}{u_{01}}\right)$$

where one can instantly notice that (11) is not a physical solution due to the condition of negative *n_l_*. As for (12), in the case of the fiber analyzed in this paper, *u_νj_* = *u*_03_ = 8.654 and *u*_01_ = 2.405, which means that *r*_2_ must be greater than 3*r*_1_. Although such NCHCFs have been presented [38], it is impossible to obtain an NCHCF-based LPG with the consecutive fiber sections differing so much in their radii. Due to the heat-based LPG inscription procedure, either by the CO_2_ laser or electrical arc discharge, the amount of microstructure collapse for the heated fiber sections would be too severe to maintain the fiber’s waveguiding properties. Of course, one can assume coupling to different high-order LP modes, with lower values of *u_νj_*. Still, even if one chooses to couple to the LP11 mode for which *u_νj_* = *u*_11_ = 3.8317 (closest to the LP01 mode), it results in *r*_2_ > 1.6*r*_1_, which is still too great a difference to obtain without damaging the NCHCF structure significantly.

#### 2.4.2. Case 2—*S*_Δ*n*NCHCF_ < 0, *S*_ΔnLPG_ < 0 and *F* > 0

The second case of Equation (9) assumes that both *S*_Δ*n*LPG_ and *S*_Δ*n*NCHCF_ are negative (i.e., their antiresonant/dip wavelengths shift towards the shorter wavelengths), which results in the following inequality when *S*_Δ*n*LPG_ is higher than *S*_Δ*n*NCHCF_:

(13) $$\frac{-4t}{\left(2m+1\right)\sqrt{n_{g}{}^{2}-n_{l}{}^{2}}}>\frac{\Lambda }{N}\left(\frac{1}{\sqrt{n_{l}{}^{2}-\frac{u_{01}{}^{2}}{k_{0}{}^{2}r_{1}{}^{2}}}}-\frac{1}{\sqrt{n_{l}{}^{2}-\frac{u_{\nu j}{}^{2}}{k_{0}{}^{2}r_{2}{}^{2}}}}\right)$$

The authors chose to solve (13) with respect to *Λ* in order to determine the direct design parameter of the LPG. The final solution takes the following form:

(14) $$\Lambda >4\sqrt{\frac{A_{1}+2\sqrt{A_{2}}+A_{3}-A_{4}+A_{5}}{A_{6}}}$$

with the *A*_1_–*A*_6_ coefficients presented in Table 2.

If (14) is satisfied, the designed LPG will have better RI sensitivity than its corresponding NCHCF. However, the determined *Λ* will also have to satisfy the LPG phase-matching condition (Equation (1)). As a result, further analysis is conducted by assuming that the grating length is equal to $4\sqrt{\frac{A_{1}+2\sqrt{A_{2}}+A_{3}-A_{4}+A_{5}}{A_{6}}}$ and then combining it with Equation (1), which results in the following inequality:

(15) $$\Lambda \left(n_{eff}^{f}-n_{eff}^{ho}\right)>\Lambda_{b}\left(n_{eff}^{f}-n_{eff}^{ho}\right),$$

where *Λ* is determined by (14) and $\Lambda_{b}=4\sqrt{\frac{A_{1}+2\sqrt{A_{2}}+A_{3}-A_{4}+A_{5}}{A_{6}}}$ is the boundary length of the LPG, for which *S*_Δ*n*NCHCF_ = *S*_Δ*n*LPG_. Additionally, from Equation (1), we know that *Λ*($n_{eff}^{f}-n_{eff}^{ho}$) = *Nλ_N_*, while *N* = 1 and λ*_N_* = 2π/*k*_0_. Thus, (15) can be further modified:

(16) $$\frac{2\pi }{k_{0}}>\Lambda_{b}\left(n_{eff}^{f}-n_{eff}^{ho}\right)\Rightarrow 2\pi >\Lambda_{b}k_{0}\left(n_{eff}^{f}-n_{eff}^{ho}\right)$$

Inequality (16) can then be considered the second condition necessary to meet in order to obtain an NCHCF-based LPG with its RI sensitivity higher than the RI sensitivity of its corresponding NCHCF. Knowing the geometrical parameters of the NCHCF (*r_F_*_1_ = 10 µm, *r_F_*_2_ = 10.4 µm and *t_F_*_1_ = 1 µm) as well as the optical parameters (*n_g_* = 1.455, *n_l_* = 1.33, *k*_0_ = 8.96957 $\times \;10^{6}\frac{1}{m}$ and *u*_01_ = 2.405), we can reduce the number of variables in $\Lambda_{b}=4\sqrt{\frac{A_{1}+2\sqrt{A_{2}}+A_{3}-A_{4}+A_{5}}{A_{6}}}$ so that only *u_νj_* remains. Substituting this “reduced” version of the *Λ_b_* formula into (16) allows the determination of the range of *u_νj_* (i.e., the range of high-order modes) for which both the (14) and (16) conditions will be met, effectively resulting in obtaining an LPG with a higher RI sensitivity than the NCHCF alone. The final results for the LPG considered in this paper are presented in the first row of Table 3. Calculated ranges of *u_νj_* indicate that the high-order mode coupling conditions are unfavorable for such grating. For the first allowed range of *u_υj_* (equal to or smaller than 2.501) there are no known LP-type modes other than the LP01, making it impossible to satisfy the coupling conditions mentioned in Section 2.3. The second range of *u_νj_* is very high and very narrow at the same time—between 122.146 and 124.067—for which there are no LP0*x*-type modes, while the closest is LP139 (*u*_139_ *=* 123.304). Unfortunately, coupling to a mode of such high order causes a major technical problem—the period of the LPG would be extremely short, in the order of single micrometers or lower (in the case of this particular mode, *Λ* ≈ 0.81 µm). In general, for the assumed NCHCF structure, one can draw a conclusion that obtaining an LPG based on such a fiber with its intended RI sensitivity higher than the sensitivity of the fiber alone is a very difficult task. It is important to remember that (14) and (16) are both multi-parameter inequalities, which can be analyzed with respect to different variables, i.e., for NCHCFs core radius, perturbed core radius, grating wavelength, etc. Such rigorous analysis is beyond the scope of this article; however, in order to gain at least a crude insight into the general behavior of (14), it was decided to solve it for different values of the following parameters: initial core radius of the base NCHCF (*r*_1_), core radius of the perturbed NCHCF region (*r*_2_), NCHCF capillary wall thickness (*t*) and the LPG wavelength (*λ*). The results are presented in Table 3 and can be easily compared with the ones obtained for the initial fiber. A general observation can be made that none of the parameters shift the ranges of *u_νj_* significantly towards the values that would result in feasible NCHCF-based LPGs. A simultaneous increase in *r*_1_ and *r*_2_ shifts the second range of allowed *u_νj_* even further towards high values, which will cause the grating lengths to be even shorter. By increasing only the *r*_2_, the values from the first range of allowed *u_υj_* also rise, but the closest possible LP-type mode in this range is LP11, for which *u_υj_* = 3.832 and corresponds to *r*_2_ ≈ 16 μm, effectively leading to the same conclusions as described previously in Section 2.4.1. Reduction of *t* expands the second range of possible *u_νj_* towards lower values, but the expansion is relatively small (from 122.146 to 121.692 for *t* = 1 and 0.9 µm, respectively). Additionally, further reduction of *t* will influence the $\lambda_{m}^{\left(\mathrm{AR}\right)}$, causing the grating’s wavelength to be also shifted in order to match the fiber’s transmission window and increasing the range of *u_νj_*. The only omitted parameter was *m*, since its increase shifts the $\lambda_{m}^{\left(\mathrm{AR}\right)}$ further towards shorter wavelengths and is likely to cause the shifted transmission window to appear in the UV region, possibly even below 250 nm, which is unfavorable not only for the aqueous samples but for the NCHCF as well. Overall, based on the analysis and presented results, an observation can be made that the calculated differences in the NCHCFs’ and NCHCF-based LPGs’ refractive index sensitivities are of a more general character.

## 3. Summary and Conclusions

The presented results constitute one of the first numerical analyses of the topic of long-period gratings written on negative curvature hollow-core fibers, addressing the matter of their possible use in sensing the refractive index changes in liquid samples. NCHCFs are, in general, considered a good medium to fill with liquids, not only because of their unique optical properties, but also due to the ease of the procedure, as the diameters of their core and capillaries are relatively large and do not pose a challenge in the filling procedure. The potential of these fibers for refractive index sensing was shown by analyzing two fibers with the well-known revolver geometry, consisting of six capillaries surrounding the core, with the core and capillary wall diameters being *D_F_*_1_ = 20 µm, *t_F_*_1_ = 1 µm and *D_F_*_2_ = 20.8 µm, *t_F_*_2_ = 1.1 µm for the two investigated fibers—F1 and F2. Small differences in the diameters reflected the changes expected to appear during the LPG-inscription procedure, which is usually some kind of heat processing—either with a CO_2_ laser or electrical arc discharge. The responses of both fibers to the change in their inner refractive index (ranging from 1.33 to 1.42) were observed as a significant shift in their antiresonant wavelengths, resulting in the calculated RI sensitivities being as high as *S*_Δ*nF*1_ = −3744 nm/RIU and *S*_Δ*nF*2_ = −4411 nm/RIU, showing another promising application of NCHCFs. An intuitive approach to the obtained results would be that by inscribing an LPG onto NCHCFs, one should obtain an even more sensitive RI sensor, since LPGs are also known to respond to the RI change with a shift in their own transmission dip. Additionally, inscribing an LPG onto NCHCFs should be relatively straightforward, although requiring additional care in order to avoid severe collapse of their microstructure. Combining the information above, a numerical model of an NCHCF-based LPG was made, using the altering segments of previously analyzed fibers, F1 and F2. The grating period *Λ* = 340 µm allowed the positioning of the LPG transmission dip at *λ*_LPG_ = 700.5 nm, and by calculating the transmission spectra of the grating for three different values of the inner refractive index (1.33, 1.36 and 1.39), the shift in the dip was observed and compared to the shift in the transmission windows of the NCHCFs. The calculated RI sensitivity was *S*_Δ*n*LPG_ = −658 nm/RIU, which is over five-times smaller than the corresponding RI sensitivity of the NCHCFs in the same RI range (−3150 and −3800 nm/RIU for fibers F1 and F2, respectively). This somewhat unexpected result was further supported by an analytical comparison of the sensitivity formulas of NCHCFs and NCHCF-based LPGs, which allows us to conclude that preparing an NCHCF-based LPG with a sensitivity higher than a bare NCHCF will be a very difficult task. Of course, the obtained results should be confirmed experimentally, especially when one considers the fact that currently available glass processing stations allow the introduction of almost any kind of point-heat-induced modifications to a fiber. Additionally, the presented analytical approach is an approximation and does not consider the mode self-coupling coefficients, and the authors believe that by including these, the analysis would become more complete, giving a better insight into the matter of propagation in this type of LPG. Finally, even though the application of NCHCF-based LPGs for RI sensing becomes questionable in light of the results presented above, the idea of such gratings should not be overlooked. Their application as, for example, in-line, narrow-band optical fiber filters is still a possibility, while the technology of heat-induced post-processing of NCHCFs is a very interesting concept in general.

## Figures and Tables

**Table 1:** Description of different cases of Equation (9).

Case No.	*S*_Δ*n*NCHCF_	*S*_Δ*n*LPG_	*F*
Case 1	*S*_Δ*n*NCHCF_ < 0	*S*_Δ*n*LPG_ > 0	*F* < 2*S*_Δ*n*NCHCF_
Case 2		*S*_Δ*n*LPG_ < 0	*F* > 0

Case 1 is equal to the transmission dips of the LPG shifting in the direction opposite to the shift of the resonant wavelengths of the NCHCF. Case 2 means that both *S*_Δ*n*LPG_ < 0 and *S*_Δ*n*NCHCF_ < 0, which demonstrates that the shift in both LPG and NCHCF occurs in the same direction (towards the shorter wavelengths). Because the two cases require different approaches, we will discuss them separately in the following sections. All the calculations presented below were conducted and analyzed by means of the Wolfram^®^ Mathematica software.

**Table 2:** Coefficients of the inequality (14).

Coefficient No.
$A_{1}=2{\left(1+2m\right)}^{2}t^{2}k_{0}^{8}n_{l}^{6}\left(n_{g}^{2}-n_{l}^{2}\right)N^{2}r_{1}^{4}r_{2}^{4}$
$A_{2}={\left(1+2m\right)}^{4}t^{4}k_{0}^{4}{\left(n_{g}^{2}-n_{l}^{2}\right)}^{2}N^{4}r_{1}^{2}r_{2}^{2}{\left(k_{0}^{2}n_{l}^{2}r_{1}^{2}-u_{1}^{2}\right)}^{3}{\left(k_{0}^{2}n_{l}^{2}r_{2}^{2}-u_{\nu j}^{2}\right)}^{3}$
$A_{3}=3{\left(1+2m\right)}^{2}t^{2}k_{0}^{6}n_{l}^{4}\left(-n_{g}^{2}+n_{l}^{2}\right)N^{2}r_{1}^{2}r_{2}^{2}\left(r_{2}^{2}u_{1}^{2}+r_{1}^{2}u_{\nu j}^{2}\right)$
$A_{4}={\left(1+2m\right)}^{2}t^{2}k_{0}^{2}\left(n_{g}^{2}-n_{l}^{2}\right)N^{2}u_{1}^{2}u_{\nu j}^{2}\left(r_{2}^{2}u_{1}^{2}+r_{1}^{2}u_{\nu j}^{2}\right)$
$A_{5}={\left(1+2m\right)}^{2}t^{2}k_{0}^{4}n_{l}^{2}\left(n_{g}^{2}-n_{l}^{2}\right)N^{2}\left(r_{2}^{4}u_{1}^{4}+4r_{1}^{2}r_{2}^{2}u_{1}^{2}u_{\nu j}^{2}+r_{1}^{4}u_{\nu j}^{4}\right)$
$A_{6}={\left(1+2m\right)}^{4}k_{0}^{4}{\left(n_{g}^{2}-n_{l}^{2}\right)}^{2}{\left(r_{2}^{2}u_{1}^{2}-r_{1}^{2}u_{\nu j}^{2}\right)}^{2}$

**Table 3:** Solutions of inequality (16) (*u_νj_*) for different NCHCF and LPG parameters. The values in bold are changed in comparison to the parameters of the initial NCHCF and LPG, which are presented in the first row of the table. In the case of *t* = 0.8 µm, the *λ*_LPG_ has also been changed to match the shifted first-order antiresonant bands of both NCHCFs. The antiresonance order and grating wavelength order assumed for the calculations are *m* = 1 and *N* = 1, respectively.

*r*_1_ (µm)	*r*_2_ (µm)	*t* (µm)	*λ*_LPG_ (nm)	*u_νj_*
10	10.4	1	700.5	(0 ≤ *u_νj_* < 2.501) or (122.146 < *u_νj_* ≤ 124.067)
**15**	**15.4**	1	700.5	(0 ≤ *u_νj_* < 2.469) or (180.871 < *u_νj_* ≤ 183.715)
**20**	**20.4**	1	700.5	(0 ≤ *u_νj_* ≤ 2.453) or (239.596 < *u_νj_* ≤ 243.362)
10	**11**	1	700.5	(0 ≤ *u_νj_* < 2.645) or (129.193 < *u_νj_* ≤ 131.225)
10	**12**	1	700.5	(0 ≤ *u_νj_* < 2.886) or (140.938 < *u_νj_* ≤ 143.154)
10	10.4	**0.9**	700.5	(0 ≤ *u_νj_* < 2.501) or (121.692 < *u_νj_* ≤ 124.067)
10	10.4	**0.8**	**634.0**	(0 ≤ *u_νj_* < 2.501) or (134.313 < *u_νj_* ≤ 137.080)

**Figure A1:**
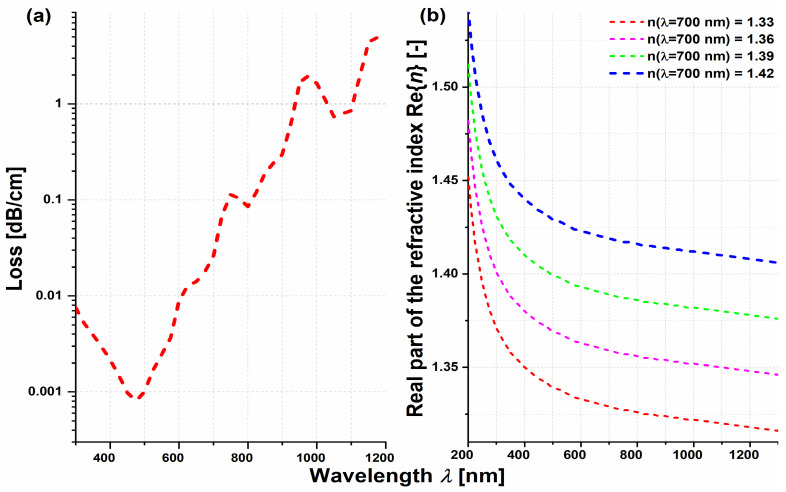
Loss spectra of pure water (**a**) and dispersion curves of aqueous sample models (**b**). The dispersion curves were calculated based on the real part of the refractive index of pure water (red dashed line).

## Data Availability

Data is contained within the article and Appendix A.

## Appendix A. Dispersion Curves of the Liquid Sample Models and Water Attenuation Spectrum

The sensor is assumed to be filled with samples containing mostly water, mixed with an unspecified composition of organic compounds, characteristic of biochemical samples. As a result, the authors assumed that liquids with *n* = 1.36, 1.39 and 1.42 (at λ ≈ 700 nm) will preserve the character of the dispersion curve of water. The only change will appear in the values of their refractive indices (real parts), which will be shifted by a constant value of Δ*n*, corresponding to each value of *n* as follows:

$$\Delta n=\{\begin{array}{c} 0.03\;\mathrm{for}\;n=1.36\\ 0.06\;\mathrm{for}\;n=1.39\\ 0.09\;\mathrm{for}\;n=1.42 \end{array}$$

The refractive index curves shifted in this manner are presented in Figure A1a. Additionally, in order to allow for a convenient comparison of the shape of designed fibers’ and gratings’ transmission and loss spectra, in Figure A1b, the water loss spectrum (in dB/cm) is also presented. The loss was calculated based on the imaginary part of the refractive index model of water from the Lumerical^®^ materials library (Palik H_2_O model).
